# Supplementary material for: Adjuvanted RSVPreF3 vaccine impact over three respiratory syncytial virus seasons in older adults with comorbidities
Source: ERJ Open Res. 2026 Sep 21;12(5):00418-2026. doi: 10.1183/23120541.00418-2026 (PMC13591300; doi:10.1183/23120541.00418-2026)
Supplement: Supplementary file 1 [file 00418-2026.SUPPLEMENT.pdf]

Supplementary material

Adjuvanted RSVPreF3 vaccine impact over 3 RSV seasons in older adults with comorbidities

Alberto Papi, David M. G. Halpin, Robert G. Feldman, Michael G. Ison, Tino F. Schwarz, Dong-Gun Lee, Raffaele Antonelli Incalzi, Laurence Fissette, Stebin Xavier, Marie-Pierre David, Jean-Philippe Michaud, Shady Kotb, Céline Maréchal, Aurélie Olivier, Veronica Hulstrøm, Marie Van der Wielen, on behalf of the AReSVi-006 study group

Supplementary methods

Symptoms and signs used to define acute respiratory illness (ARI) and lower respiratory tract disease (LRTD)

Respiratory symptoms were: nasal congestion/rhinorrhoea; sore throat; new or increased sputum (lower respiratory symptom); new or increased cough (lower respiratory symptom); new or increased dyspnoea (shortness of breath) (lower respiratory symptom).

Respiratory signs were: new or increased wheezing (reported by the participant or investigator) (lower respiratory sign); new or increased crackles/rhonchi (reported by the investigator) based on chest auscultation (lower respiratory sign); respiratory rate ≥20 respirations/min (reported by the investigator) (lower respiratory sign); low or decreased oxygen saturation (= oxygen saturation <95% or ≤90% if pre-season baseline was <95%, reported by the investigator) (lower respiratory sign); need for oxygen supplementation (reported by the investigator) (lower respiratory sign).

Systemic symptoms/signs were: fever (body temperature ≥38.0°C/100.4°F by any route) or feverishness (feeling of having fever without objective measurement); fatigue; body aches; headache; decreased appetite.

An LRTD was classified as severe (1) if the episode was assessed as severe by the investigator or if the participant experienced at least two lower respiratory signs (as listed above), or (2) if the participant required oxygen supplementation, positive airway pressure therapy, or other types of mechanical ventilation, or any significant change or adaptation in one of these therapies if already used [1].

Complications

The following complications were collected throughout the study, starting on day 1.

<u>Respiratory complications:</u>

- Pneumonia: a clinical diagnosis of pneumonia based on signs and symptoms, with or without chest radiograph that demonstrated a new or progressive infiltrate.
- New diagnosis of chronic obstructive pulmonary disease (COPD) or exacerbation of COPD: a new diagnosis of COPD or, in a participant with previously diagnosed COPD, a worsening of COPD necessitating an increase in the dosage or modification of the administered treatment.
- New diagnosis of asthma or exacerbation of asthma: a new diagnosis of asthma or, in a participant with previously diagnosed asthma, a worsening of asthma necessitating an increase in the dosage or modification of the administered treatment.
- Other respiratory complications: other diagnosis of respiratory illness, including new diagnosis or exacerbation of a pre-existing respiratory disease (e.g., emphysema, chronic bronchitis).

Non-respiratory complications:

- New onset or worsening of congestive heart failure
- Myocardial infarction
- Stroke
- Diabetes
- Other non-respiratory complications judged related to an ARI episode by the investigator

Immunogenicity assessments

Respiratory syncytial virus (RSV)-A and RSV-B neutralising titres were measured (as described previously [1]) in a subset of participants from a selected number of countries and trial sites (immunogenicity cohort) using serum samples collected on day 1 (prior to dose 1), day 31, pre-season 2 (prior to dose 2), and pre-season 3.

Immunogenicity was evaluated in the per-protocol population (protocol-compliant participants in the immunogenicity cohort who had post-dose immunogenicity data available). RSV-A and RSV-B neutralising geometric mean titres (GMTs) and mean geometric increases (MGIs) were calculated with 95% confidence intervals in subgroups of participants without any of the pre-existing conditions of interest, with ≥1 condition, ≥1 cardiorespiratory, and ≥1 endocrine or metabolic condition.

Supplementary results

Efficacy of revaccination regimen against RSV-LRTD and RSV-ARI

The cumulative efficacy of the revaccination regimen (two doses given approximately 1 year apart) over three RSV seasons post-dose 1 was generally in the same range as that of a single vaccine dose among the different subgroups of participants with pre-existing medical conditions, although point estimates for the revaccination regimen were numerically higher for most subgroups (**Supplementary Table 5**).

Immune response and persistence in participants with pre-existing medical conditions

Regardless of the presence of pre-existing medical conditions of interest, RSV-A and RSV-B neutralising GMTs increased from pre-vaccination to day 31, with MGIs versus pre-vaccination ranging from 9.4 to 11.7 for RSV-A and 8.1 to 9.3 for RSV-B across groups (RSV revaccination and RSV single-dose) and subgroups (no condition of interest, ≥1 condition, ≥1 cardiorespiratory condition, ≥1 endocrinometabolic condition) (**Supplementary Figure 1**). Neutralising GMTs decreased over time but remained above baseline levels until at least pre-RSV season 3 irrespective of the vaccination schedule and the presence of pre-existing conditions, with MGIs versus pre-vaccination ranging from 2.2 to 3.5 for RSV-A and 1.6 to 2.4 for RSV-B across groups and subgroups (**Supplementary Figure 1**).

Supplementary Table 1. Vaccine efficacy of a single adjuvanted RSVPreF3 dose against first occurrence of severe RSV-LRTD over three RSV seasons, overall and among participants with pre-existing medical conditions (modified exposed population)

Endpoint	Adjuvanted RSVPreF3 group				Placebo group				Vaccine efficacy,
Subgroup	N	n	T, py	IR per 1000 py	N	n	T, py	IR per 1000 py	% (95% CI)
**Severe RSV-LRTD**[a]									
Overall	12,468	15	19,778.4	0.8	12,498	75	27,526.4	2·7	67.4 (42.4–82.7)
By pre-existing condition of interest[b]									
No condition	7454	7	11,828.1	0.6	7547	30	16,711.0	1.8	63.5 (14.3–86.6)
≥1 condition	5014	8	7950.3	1.0	4951	45	10,815.4	4.2	70.7 (36.9–88.1)
≥1 cardiorespiratory condition	2577	6	4059.0	1.5	2504	39	5441.5	7.2	73.8 (37.4–91.0)
≥1 endocrine/metabolic condition	3243	4	5159.6	0.8	3274	15	7189.2	2.1	57.2 (−36.2–89.8)

Results based on post-hoc exploratory analyses, except for those in the overall trial population. Analysis included data collected from day 15 post-dose 1 until end of Northern Hemisphere season 3 (efficacy data lock point: 30 April 2024) or until end of Southern Hemisphere season 1 (efficacy data lock point: 30 September 2022) for participants who did not receive dose 2. Participants who received adjuvanted RSVPreF3 as dose 1 and placebo as dose 2 (RSV single-dose group) and participants in the placebo group contributed to the entire follow-up. Participants who received adjuvanted RSVPreF3 as doses 1 and 2 (RSV revaccination group) contributed to season 1 but were censored at dose 2. Vaccine efficacy was estimated using a Poisson model adjusted for age, region, and season.

Adjuvanted RSVPreF3, adjuvanted respiratory syncytial virus (RSV) prefusion F protein-based vaccine; RSV-LRTD, RSV-related lower respiratory tract disease confirmed by the adjudication committee; N, number of participants in the modified exposed population; n, number of participants with ≥1 severe RSV-LRTD; T, sum of follow-up time from day 15 post-dose 1 until first occurrence of the event, data lock point, or drop-out; IR, incidence rate of participants reporting ≥1event, expressed per 1000 person-years (py), calculated as 1000 x (n/T); CI, confidence interval.

[a]Severe disease based on clinical signs or investigator assessment or on the need for supportive therapy. [b]More than one type of pre-existing condition of interest could occur in a single participant.

Supplementary Table 2. Vaccine efficacy of a single adjuvanted RSVPreF3 dose against first occurrence of RSV-LRTD and RSV-ARI during the first RSV season, overall and among participants with pre-existing medical conditions (modified exposed population)

Endpoint	Adjuvanted RSVPreF3 group				Placebo group				Vaccine efficacy,
Subgroup	N	n	T, py	IR per 1000 py	N	n	T, py	IR per 1000 py	% (CI[a])
**RSV-LRTD**									
Overall	12,466	7	6865.9	1.0	12,494	40	6857.3	5.8	82.6 (57.9–94.1)
By pre-existing condition of interest[b]									
COPD	1131	1	644.9	1.6	1113	8	619.6	12.9	88.1 (11.2–99.73)
Asthma	1193	0	679.3	0.0	1112	4	629.2	6.4	100 (-5.5–100)
Chronic respiratory/pulmonary disease	2223	1	1260.6	0.8	2122	12	1190.2	10.1	92.2 (47.3–99.8)
Chronic heart failure	398	0	219.6	0.0	403	2	220.5	9.1	100 (-264.0–100)
Diabetes mellitus type 1 or 2	2829	0	1589.8	0.0	2875	12	1603.5	7.5	100 (71.4–100)
Advanced liver or renal disease	667	0	370.7	0.0	674	2	380.0	5.3	100 (-257.2–100)
By BMI category[c]									
BMI 18.5–<25 kg/m^2	3051	2	1638.5	1.2	2997	5	1607.1	3.1	60.3 (-143.1–96.2)
BMI 25–<30 kg/m^2	4556	5	2511.2	2.0	4599	13	2521.6	5.2	61.3 (-15.7–89.2)
BMI ≥30 kg/m^2	4718	0	2648.7	0.0	4744	22	2655.7	8.3	100 (85.5–100)

**RSV-ARI**									
Overall	12,466	27	6858.7	3.9	12,494	95	6837.8	13.9	71.7 (56.2–82.3)
By pre-existing condition of interest[b]									
COPD	1131	1	644.9	1.6	1113	16	616.9	25.9	94.0 (61.4–99.9)
Asthma	1193	1	678.8	1.5	1112	8	628.0	12.7	88.2 (11.9–99.7)
Chronic respiratory/pulmonary disease	2223	2	1260.0	1.6	2122	22	1186.9	18.5	91.4 (65.0–99.0)
Chronic heart failure	398	1	219.1	4.6	403	4	219.8	18.2	75.2 (-150.6–99.5)
Diabetes mellitus type 1 or 2	2829	6	1587.3	3.8	2875	28	1598.2	17.5	78.4 (46.9–92.7)
Advanced liver or renal disease	667	0	370.7	0.0	674	5	378.8	13.2	100 (19.5–100)
By BMI category[c]									
BMI 18.5–<25 kg/m^2	3051	8	1636.3	4.9	2997	19	1602.0	11.9	58.1 (-0.2–84.2)
BMI 25–<30 kg/m^2	4556	13	2508.4	5.2	4599	29	2516.1	11.5	54.8 (10.3–78.5)
BMI ≥30 kg/m^2	4718	5	2646.5	1.9	4744	47	2646.8	17.8	89.4 (73.5–96.7)

Results based on post-hoc exploratory analyses, except for those in the overall trial population. Analysis included cases collected from day 15 post-dose 1 until end of Northern Hemisphere season 1 (efficacy data lock point: 11 April 2022). Vaccine efficacy was estimated using a Poisson model adjusted for age and region.

Adjuvanted RSVPreF3, adjuvanted respiratory syncytial virus (RSV) prefusion F protein-based vaccine; RSV-LRTD, RSV-related lower respiratory tract disease confirmed by the adjudication committee; RSV-ARI, RSV-related acute respiratory illness; N, number of participants in the modified exposed population; n, number of participants with ≥1 RSV-LRTD or RSV-ARI; T, sum of follow-up time from day 15 post-dose 1 until first occurrence of the event, data

lock point, or drop-out; IR, incidence rate of participants reporting at least one event, expressed per 1000 person-years (py), calculated as 1000 x (n/T); CI, confidence interval; COPD, chronic obstructive pulmonary disease; BMI, body mass index (18.5–<25 kg/m^2: healthy weight; 25–<30 kg/m^2: overweight; ≥30 kg/m^2: obesity).

[a]96.95% CI for RSV-LRTD, overall (confirmatory secondary endpoint); 95% CI for other endpoints. [b]More than one type of pre-existing condition of interest could occur in a single participant. [c]Among the 131 (adjuvanted RSVPreF3) and 145 (placebo) participants with a BMI <18.5 kg/m^2, none reported an RSV-LRTD and one (adjuvanted RSVPreF3) reported an RSV-ARI.

Supplementary Table 3. Vaccine efficacy of a single adjuvanted RSVPreF3 dose against first occurrence of RSV-LRTD and RSV-ARI during the second RSV season, overall and among participants with pre-existing medical conditions (dose 2 modified exposed population)

Endpoint<br>Subgroup	Adjuvanted RSVPreF3 group (single dose)				Placebo group				Vaccine efficacy, % (95% CI)
	N	n	T, py	IR per 1000 py	N	n	T, py	IR per 1000 py	
**RSV-LRTD**									
Overall	4991	20	2448.8	8.2	10,031	91	4914.5	18.5	56.1 (28.2–74.4)
By pre-existing condition of interest[a]									
COPD	454	1	231.7	4.3	868	17	431.7	39.4	88.3 (25.0–99.7)
Asthma	485	6	237.7	25.2	929	24	455.8	52.7	53.0 (-18.2–84.3)
Chronic respiratory/pulmonary disease	889	7	442.6	15.8	1720	38	850.6	44.7	66.0 (22.8–87.2)
Chronic heart failure	162	0	81.9	0.0	312	2	159.7	12.5	100 (-438.1–100)
Diabetes mellitus type 1 or 2	1141	6	570.2	10.5	2295	16	1146.9	14.0	25.7 (-99.9–76.2)
Advanced liver or renal disease	284	1	147.3	6.8	560	3	290.0	10.3	36.5 (-698.1–98.8)
By BMI category[b]									
BMI 18.5–<25 kg/m^2	1193	3	582.8	5.1	2402	17	1179.0	14.4	63.3 (-27.2–93.1)

BMI 25–<30 kg/m^2	1842	10	918.7	10.9	3705	31	1837.8	16.9	36.3 (-33.5–72.1)
BMI ≥30 kg/m^2	1901	7	923.9	7.6	3809	43	1848.3	23.3	68.2 (28.6–87.9)
**RSV-ARI**									
Overall	4991	54	2438.1	22.1	10,031	181	4888.0	37.0	40.6 (19.0–57.0)
By pre-existing condition of interest[a]									
COPD	454	4	230.5	17.4	868	24	429.6	55.9	66.9 (3.2–91.7)
Asthma	485	9	236.8	38.0	929	27	454.4	59.4	37.5 (-37.2–74.2)
Chronic respiratory/pulmonary disease	889	13	440.5	29.5	1720	49	846.7	57.9	50.0 (6.2–75.2)
Chronic heart failure	162	1	81.9	12.2	312	6	158.9	37.7	72.1 (-131.9–99.4)
Diabetes mellitus type 1 or 2	1141	10	568.8	17.6	2295	34	1142.0	29.8	41.4 (-21.4–74.2)
Advanced liver or renal disease	284	5	145.8	34.3	560	7	289.1	24.2	-34.5 (-394.4–66.5)

Results based on post-hoc exploratory analyses, except for those in the overall trial population. Analysis included cases collected from day 15 post-dose 2 until end of Northern Hemisphere season 2 (efficacy data lock point: 31 March 2023) from participants who received adjuvanted RSVPreF3 as dose 1 and placebo as dose 2 and from participants who received placebo as doses 1 and 2. Vaccine efficacy was estimated using a Poisson model adjusted for age and region.

Adjuvanted RSVPreF3, adjuvanted respiratory syncytial virus (RSV) prefusion F protein-based vaccine; RSV-LRTD, RSV-related lower respiratory tract disease confirmed by the adjudication committee; RSV-ARI, RSV-related acute respiratory illness; N, number of participants in the dose 2 modified exposed population; n, number of participants with ≥1 RSV-LRTD or RSV-ARI; T, sum of follow-up time from day 15 post-dose 2 until first occurrence of the event, data lock point, or drop-out; IR, incidence rate of participants reporting at least one event, expressed per 1000 person-years (py), calculated as 1000 x (n/T); CI,

confidence interval; COPD, chronic obstructive pulmonary disease; BMI, body mass index (18.5–<25 kg/m^2: healthy weight; 25–<30 kg/m^2: overweight; ≥30 kg/m^2: obesity).

[a]More than one type of pre-existing condition of interest could occur in a single participant. [b]Among the 50 (adjuvanted RSVPreF3) and 112 (placebo) participants with a BMI <18.5 kg/m^2, none reported an RSV-LRTD.

Supplementary Table 4. Vaccine efficacy of a single adjuvanted RSVPreF3 dose against first occurrence of RSV-LRTD and RSV-ARI during the third RSV season, overall and among participants with pre-existing medical conditions (dose 2 modified exposed population)

Endpoint Subgroup	Adjuvanted RSVPreF3 group (single dose)				Placebo group				Vaccine efficacy, % (95% CI)
	N	n	T, py	IR per 1000 py	N	n	T, py	IR per 1000 py	
**RSV-LRTD**									
Overall	4988	16	2717.2	5.9	10,031	61	5407.4	11.3	48.0 (8.7–72.0)
By pre-existing condition of interest[a]									
COPD	464	3	245.4	12.2	881	10	457.9	21.8	40.1 (-134.6–89.5)
Asthma	494	2	270.2	7.4	946	14	508.9	27.5	72.1 (-21.6–96.9)
Chronic respiratory/pulmonary disease	903	5	484.3	10.3	1744	23	921.6	25.0	58.1 (-12.8–87.6)
Chronic heart failure	166	0	81.7	0.0	317	1	167.1	6.0	100 (-3879.3–100)
Diabetes mellitus type 1 or 2	1144	2	613.1	3.3	2304	16	1227.5	13.0	74.8 (-7.3–97.2)
Advanced liver or renal disease	289	2	149.9	13.3	573	4	305.1	13.1	-2.4 (-620.4–90.8)
By BMI category[b]									
BMI 18.5–<25 kg/m^2	1192	2	650.8	3.1	2402	8	1294.8	6.2	50.7 (-147.5–94.9)

BMI 25–<30 kg/m^2	1842	6	1012.3	5.9	3705	21	2008.7	10.5	43.3 (-45.3–81.3)
BMI ≥30 kg/m^2	1899	8	1024.9	7.8	3809	32	2044.9	15.6	50.4 (-10.0–80.3)
**RSV-ARI**									
Overall	4988	30	2712.6	11.1	10,031	113	5391.5	21.0	47.4 (20.7–66.1)
By pre-existing condition of interest[a]									
COPD	464	3	245.4	12.2	881	13	457.1	28.4	55.1 (-64.6– 91.8)
Asthma	494	5	269.1	18.6	946	18	507.7	35.5	45.1 (-53.8–84.1)
Chronic respiratory/pulmonary disease	903	8	483.2	16.6	1744	29	919.9	31.5	47.2 (-18.6–79.2)
Chronic heart failure	166	1	81.4	12.3	317	2	166.8	12.0	-11.1 (-2042.8–98.1)
Diabetes mellitus type 1 or 2	1144	4	612.7	6.5	2304	24	1224.8	19.6	66.7 (3.1–91.6)
Advanced liver or renal disease	289	2	149.9	13.3	573	8	303.9	26.3	50.3 (-150.5–94.9)
By BMI category[b]									
BMI 18.5–<25 kg/m^2	1192	8	648.8	12.3	2402	23	1290.1	17.8	30.4 (-61.3–73.1)
BMI 25–<30 kg/m^2	1842	11	1010.5	10.9	3705	43	2001.7	21.5	50.0 (1.3–76.7)
BMI ≥30 kg/m^2	1899	11	1024.1	10.7	3809	45	2041.1	22.0	50.8 (3.3–77.1)

Results based on post-hoc exploratory analyses, except for those in the overall trial population. Analysis included cases collected from the start of Northern Hemisphere season 3 (1 October 2023) until the end of Northern Hemisphere season 3 (efficacy data lock point: 30 April 2024) from participants who received adjuvanted RSVPreF3 as dose 1 and placebo as dose 2 and from participants who received placebo as doses 1 and 2. Vaccine efficacy was estimated using a Poisson model adjusted for age and region.

Adjuvanted RSVPreF3, adjuvanted respiratory syncytial virus (RSV) prefusion F protein-based vaccine; RSV-LRTD, RSV-related lower respiratory tract disease confirmed by the adjudication committee; RSV-ARI, RSV-related acute respiratory illness; N, number of participants in the dose 2 modified exposed population; n, number of participants with ≥1 RSV-LRTD or RSV-ARI; T, sum of follow-up time from start of season 3 until first occurrence of the event, data lock point, or drop-out; IR, incidence rate of participants reporting at least one event, expressed per 1000 person-years (py), calculated as 1000 x (n/T); CI, confidence interval; COPD, chronic obstructive pulmonary disease; BMI, body mass index (18.5–<25 kg/m^2: healthy weight; 25–<30 kg/m^2: overweight; ≥30 kg/m^2: obesity).

[a]More than one type of pre-existing condition of interest could occur in a single participant. [b]Among the 50 (adjuvanted RSVPreF3) and 112 (placebo) participants with a BMI <18.5 kg/m^2, none reported an RSV-LRTD and two (placebo) reported an RSV-ARI.

Supplementary Table 5. Vaccine efficacy of a first adjuvanted RSVPreF3 dose followed by a second dose 1 year later (revaccination) against first occurrence of RSV-LRTD and RSV-ARI over three RSV seasons, overall and among participants with pre-existing medical conditions (modified exposed population)

Endpoint	Adjuvanted RSVPreF3 (revaccination)				Placebo				Vaccine efficacy, %
Subgroup	N	n	T, py	IR per 1000 py	N	n	T, py	IR per 1000 py	(CI[a])
**RSV-LRTD**									
Overall	12,468	38	18,744.4	2.0	12,498	192	25,468.3	7.5	67.8 (51.8–79.1)
By pre-existing condition of interest[b]									
No condition	7454	20	11,283.8	1.8	7547	91	15,558.9	5.8	64.2 (41.1–79.2)
≥1 condition	5014	18	7460.6	2.4	4951	101	9909.4	10.2	71.2 (51.9–83.7)
≥1 cardiorespiratory condition	2577	11	3793.9	2.9	2504	73	4964.0	14.7	75.9 (54.1–88.5)
COPD	1181	3	1720.6	1.7	1161	35	2265.0	15.5	86.1 (55.5–97.3)
Asthma	1226	9	1836.6	4.9	1160	41	2372.5	17.3	63.8 (23.8–84.7)
Chronic respiratory/pulmonary disease	2287	11	3391.9	3.2	2200	72	4405.8	16.3	75.7 (53.7–88.5)
Chronic heart failure	427	0	585.4	0.0	425	4	782.4	5.1	100.0 (-53.7–100.0)
≥1 endocrine/metabolic condition	3243	9	4846.6	1.9	3274	49	6590.0	7.4	69.9 (37.5–87.1)
Diabetes mellitus type 1 or 2	2856	8	4276.4	1.9	2898	44	5831.9	7.5	70.5 (36.2–88.1)
Advanced liver or renal disease	698	1	1021.6	1.0	710	10	1426.4	7.0	82.1 (-29.2–99.6)

By BMI category[c]									
BMI 18.5–<25 kg/m^2	3051	7	4602.4	1.5	2999	32	6127.3	5.2	63.9 (16.1–86.6)
BMI 25–<30 kg/m^2	4560	21	6873.7	3.1	4600	63	9356.9	6.7	45.1 (8.2–68.3)
BMI ≥30 kg/m^2	4716	10	7071.9	1.4	4745	97	9689.9	10.0	83.2 (67.6–92.2)
**RSV-ARI**									
Overall	12,468	103	18.678.5	5.5	12,498	385	25,236.9	15.3	58.4 (48.1–67.0)
By pre-existing condition of interest[b]									
No condition	7454	63	11,238.5	5.6	7547	212	15,411.4	13.8	53.4 (37.9–65.5)
≥1 condition	5014	40	7440.0	5.4	4951	173	9825.5	17.6	64.6 (49.7–75.7)
≥1 cardiorespiratory condition	2577	21	3785.3	5.5	2504	106	4924.5	21.5	69.7 (51.0–82.1)
COPD	1181	5	1719.4	2.9	1161	50	2242.9	22.3	85.1 (62.6–95.4)
Asthma	1226	16	1830.0	8.7	1160	53	2357.3	22.5	51.3 (12.8–74.2)
Chronic respiratory/pulmonary disease	2287	20	3384.1	5.9	2200	98	4372.4	22.4	68.5 (48.3–81.6)
Chronic heart failure	427	1	584.6	1.7	425	11	776.2	14.2	87.1 (8.0–99.7)
≥1 endocrine/metabolic condition	3243	23	4833.5	4.8	3274	100	6530.9	15.3	65.0 (44.3–78.9)
Diabetes mellitus type 1 or 2	2856	21	4263.7	4.9	2898	89	5776.5	15.4	64.5 (42.0–79.1)
Advanced liver or renal disease	698	3	1021.1	2.9	710	25	1412.9	17.7	80.3 (34.5–96.2)
By BMI category[c]									
BMI 18.5–<25 kg/m^2	3051	29	4581.7	6.3	2999	83	6066.1	13.7	46.1 (16.5–66.1)

BMI 25–<30 kg/m^2	4560	42	6850.1	6.1	4600	133	9278.4	14.3	49.9 (28.3–65.6)
BMI ≥30 kg/m^2	4716	30	7050.7	4.3	4745	167	9598.5	17.4	72.5 (59.1–82.1)

Results based on post-hoc exploratory analyses, except for those in the overall trial population, in participants with no condition, ≥1 condition, ≥1 cardiorespiratory, and ≥1 endocrine/metabolic condition of interest. Analysis included data collected from day 15 post-dose 1 until end of Northern Hemisphere season 3 (efficacy data lock point: 30 April 2024) in participants who did not receive dose 3, or until dose 3 administration, or until end of Southern Hemisphere season 1 (efficacy data lock point: 30 September 2022) for participants who did not receive dose 2. Participants who received adjuvanted RSVPreF3 as doses 1 and 2 (RSV revaccination group) and participants in the placebo group contributed to the entire follow-up. Participants who received adjuvanted RSVPreF3 as dose 1 and placebo as dose 2 (RSV single-dose group) contributed to season 1 but were censored at dose 2. Vaccine efficacy was estimated using a Poisson model adjusted for age, region, and season.

Adjuvanted RSVPreF3, adjuvanted respiratory syncytial virus (RSV) prefusion F protein-based vaccine; RSV-LRTD, RSV-related lower respiratory tract disease confirmed by the adjudication committee; RSV-ARI, RSV-related acute respiratory illness; N, number of participants in the modified exposed population; n, number of participants with ≥1 RSV-LRTD or RSV-ARI; T, sum of follow-up time (from day 15 post-dose 1 until first occurrence of the event, data lock point, drop-out, or dose 3 administration); IR, incidence rate of participants reporting at least one event, expressed per 1000 person-years (py), calculated as 1000 x (n/T); CI, confidence interval; COPD, chronic obstructive pulmonary disease; BMI, body mass index (18.5–<25 kg/m^2: healthy weight; 25–<30 kg/m^2: overweight; ≥30 kg/m^2: obesity).

[a]97.5% CI for RSV-LRTD, overall (confirmatory secondary endpoint); 95% CI for other endpoints. [b]More than one type of pre-existing condition of interest could occur in a single participant. [c]Among the 131 (adjuvanted RSVPreF3) and 145 (placebo) participants with a BMI <18.5 kg/m^2, none reported an RSV-LRTD, and two (adjuvanted RSVPreF3) and two (placebo) reported an RSV-ARI.

Supplementary Table 6. Occurrence of RSV-ARI-related complications over three RSV seasons (modified exposed population)

Complication	RSV single-dose group (N=6225)	Placebo group (N=12,498)
At least one complication	11 (0.2)	50 (0.4)
At least one respiratory complication	11 (0.2)	48 (0.4)
Pneumonia	3 (0.0)	8 (0.1)
New diagnosis of COPD	0 (0.0)	1 (0.0)
Exacerbation of COPD	3 (0.0)	13 (0.1)
New diagnosis of asthma	0 (0.0)	0 (0.0)
Exacerbation of asthma	2 (0.0)	12 (0.1)
Other respiratory complication[a]	3 (0.0)	16 (0.1)
At least one non-respiratory complication	0 (0.0)	3 (0.0)
New-onset congestive heart failure	0 (0.0)	1 (0.0)
Worsening congestive heart failure	0 (0.0)	0 (0.0)
Myocardial infarction	0 (0.0)	0 (0.0)
Stroke	0 (0.0)	0 (0.0)
Diabetes	0 (0.0)	0 (0.0)
Other non-respiratory complication[b]	0 (0.0)	2 (0.0)

Results based on post-hoc exploratory analyses. Data are n (%), with n being the number of participants reporting at least one of the indicated complications and % calculated as n/N. Analysis included data collected from day 15 post-dose 1 until end of Northern Hemisphere season 3 (efficacy data lock point: 30 April 2024) or until end of Southern Hemisphere season 1 (efficacy data lock point: 30 September 2022) for participants who did not receive dose 2.

RSV-ARI, respiratory syncytial virus (RSV)-related acute respiratory illness; N, number of participants per group in the modified exposed population; COPD, chronic obstructive pulmonary disease.

[a]Other respiratory complications reported were acute bronchitis, bacterial bronchitis, bronchitis, exacerbation of bronchiectasis, exacerbation of reactive airway disease, RSV infection, sinusitis, viral

lower respiratory tract infection, worsening of dyspnoea, worsening of viral bronchitis, whooping cough, and chest infection. [b]Other non-respiratory complications reported were acute otitis media.

Supplementary Table 7. Vaccine efficacy of a single adjuvanted RSVPreF3 dose against RSV-ARI-related complications and disease exacerbations during the first RSV season and over the first two seasons, overall and among participants with pre-existing medical conditions (modified exposed population)

Endpoint<br>Subgroup	Adjuvanted RSVPreF3 group (single dose)				Placebo group				Vaccine efficacy, % (95% CI)
	N	n	T, py	IR per 1000 py	N	n	T, py	IR per 1000 py	
**RSV season 1**									
**RSV-ARI-related complications**									
Overall	12,468	4	11,695.7	0.3	12,498	16	11,680.4	1.4	75.1 (22.7−93.9)
By pre-existing condition of interest[a]									
No condition	7454	1	6939.8	0.1	7547	5	7022.4	0.7	79.4 (-84.0−99.6)
≥1 condition	5014	3	4755.8	0.6	4951	11	4658.0	2.4	73.5 (-0.2−95.3)
≥1 cardiorespiratory condition	2577	2	2449.3	0.8	2504	11	2357.8	4.7	82.5 (20.0−98.1)
COPD	1181	0	1125.9	0.0	1161	7	1086.3	6.4	100 (47.3−100)
Asthma	1226	2	1175.2	1.7	1160	4	1109.7	3.6	53.0 (-228.3−95.8)
Chronic respiratory/pulmonary disease	2287	2	2182.3	0.9	2200	11	2081.3	5.3	82.7 (20.7−98.1)
Chronic heart failure	427	0	396.1	0.0	425	2	389.4	5.1	100 (-246.3−100)

**RSV-ARI-related exacerbation of COPD**									
By pre-existing condition of interest									
COPD	1181	0	1125.9	0.0	1161	6	1087.3	5.5	100 (37.4−100)
**RSV-ARI-related exacerbation of asthma**									
By pre-existing condition of interest									
Asthma	1226	2	1175.2	1.7	1160	3	1110.5	2.7	37.9 (-442.9−94.8)
**RSV seasons 1+ 2**									
**RSV-ARI-related complications**									
Overall	12,468	10	16,896.4	0.6	12,498	38	21,825.6	1.7	61.9 (21.4−83.2)
By pre-existing condition of interest[a]									
No condition	7454	2	10,093.8	0.2	7547	9	13,229.8	0.7	69.9 (-48.1−96.9)
≥1 condition	5014	8	6802.7	1.2	4951	29	8595.8	3.4	60.1 (9.6−84.4)
≥1 cardiorespiratory condition	2577	7	3479.7	2.0	2504	27	4328.8	6.2	63.4 (12.9−86.7)
COPD	1181	2	1597.9	1.3	1161	15	1968.0	7.6	79.9 (11.4−97.8)
Asthma	1226	5	1685.8	3.0	1160	14	2055.9	6.8	50.3 (-48.0−86.2)
Chronic respiratory/pulmonary disease	2287	7	3106.7	2.3	2200	27	3825.7	7.1	63.6 (13.3−86.8)
Chronic heart failure	427	0	555.9	0.0	425	2	700.6	2.9	100 (-245.4−100)

**RSV-ARI-related exacerbation of COPD**									
By pre-existing condition of interest									
COPD	1181	2	1597.9	1.3	1161	11	1972.2	5.6	73.9 (-23.7−97.2)
**RSV-ARI-related exacerbation of asthma**									
By pre-existing condition of interest									
Asthma	1226	3	1687.2	1.8	1160	9	2060.9	4.4	57.9 (-71.6−92.8)

Results based on post-hoc exploratory analyses, except for those in the overall trial population. Complications could include respiratory complications (pneumonia, new diagnosis or exacerbation of COPD or asthma, other respiratory complications [e.g., sinusitis, bronchitis, dyspnoea]) and non-respiratory complications (new onset or worsening of congestive heart failure, myocardial infarction, stroke, diabetes, other non-respiratory complications). Analysis for season 1 included data collected from day 15 post-dose 1 until end of Southern Hemisphere season 1 (efficacy data lock point: 30 September 2022) or up to dose 2 administration. Analysis over the first two seasons included data collected from day 15 post-dose 1 until end of Southern Hemisphere season 2 (efficacy data lock point: 30 September 2023) or up to dose 3 administration, or up to end of Southern Hemisphere season 1 (efficacy data lock point: 30 September 2022) if no dose 2 administration; participants who received adjuvanted RSVPreF3 as dose 1 and placebo as dose 2 (RSV single-dose group) and participants in the placebo group contributed to the entire follow-up; participants who received adjuvanted RSVPreF3 as doses 1 and 2 (RSV revaccination group) contributed to season 1 but were censored at dose 2. Vaccine efficacy was estimated using a Poisson model adjusted for age and region (for season 1 analysis), or age, region, and season (for analysis over two seasons).

Adjuvanted RSVPreF3, adjuvanted respiratory syncytial virus (RSV) prefusion F protein-based vaccine; RSV-ARI, RSV-related acute respiratory illness; N, number of participants in the modified exposed population; n, number of participants with ≥1 RSV-ARI-related complication; T, sum of follow-up time (from day

15 post-dose 1 until first occurrence of the event, data lock point, drop-out, or dose 2 administration [for season 1 analysis], or dose 3 administration [for analysis over two seasons]); IR, incidence rate of participants reporting at least one event, expressed per 1000 person-years (py), calculated as 1000 x (n/T); CI, confidence interval; COPD, chronic obstructive pulmonary disease.

[a]More than one type of pre-existing condition of interest could occur in a single participant.

Supplementary Figure 1. RSV-A and RSV-B neutralising titres pre-dose 1, one month post-dose 1, pre-season 2, and pre-season 3, by pre-existing medical conditions of interest (per-protocol population)

A. RSV-A neutralising titres

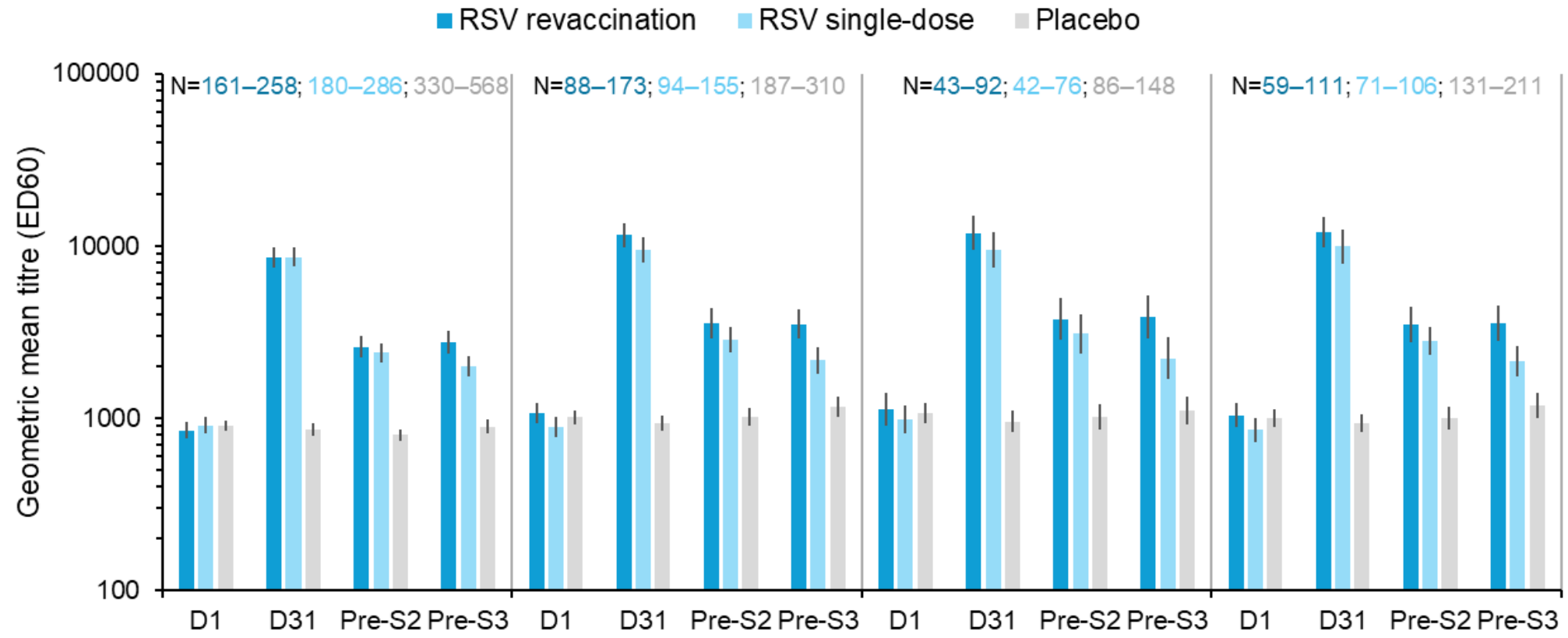

MGI vs D1 (95% CI)		No condition	≥1 condition	≥1 cardiorespiratory condition	≥1 endocrine/metabolic condition
RSV revaccination	D31	10.0 (8.9–11.4)	11.0 (9.1–13.3)	10.9 (8.4–14.2)	11.7 (9.0–15.2)
	Pre-S2	3.0 (2.7–3.4)	3.4 (2.8–4.0)	3.4 (2.7–4.4)	3.4 (2.7–4.3)
	Pre-S3	3.4 (3.1–3.8)	3.5 (2.8–4.2)	3.5 (2.6–4.8)	3.5 (2.8–4.5)
RSV single-dose	D31	9.4 (8.3–10.7)	10.8 (9.0–13.1)	9.9 (7.7–12.7)	11.6 (9.2–14.8)
	Pre-S2	2.6 (2.4–2.9)	3.1 (2.7–3.6)	3.1 (2.6–3.8)	3.2 (2.6–3.8)
	Pre-S3	2.3 (2.1–2.6)	2.4 (2.0–2.8)	2.4 (2.0–2.9)	2.2 (1.8–2.7)
Placebo	D31	1.0 (0.9–1.0)	0.9 (0.9–1.0)	0.9 (0.8–1.0)	0.9 (0.9–1.0)
	Pre-S2	0.9 (0.9–1.0)	1.0 (0.9–1.1)	1.0 (0.9–1.1)	1.0 (0.9–1.1)
	Pre-S3	1.0 (1.0–1.1)	1.1 (1.0–1.3)	1.1 (1.0–1.3)	1.2 (1.0–1.3)

B. RSV-B neutralising titres

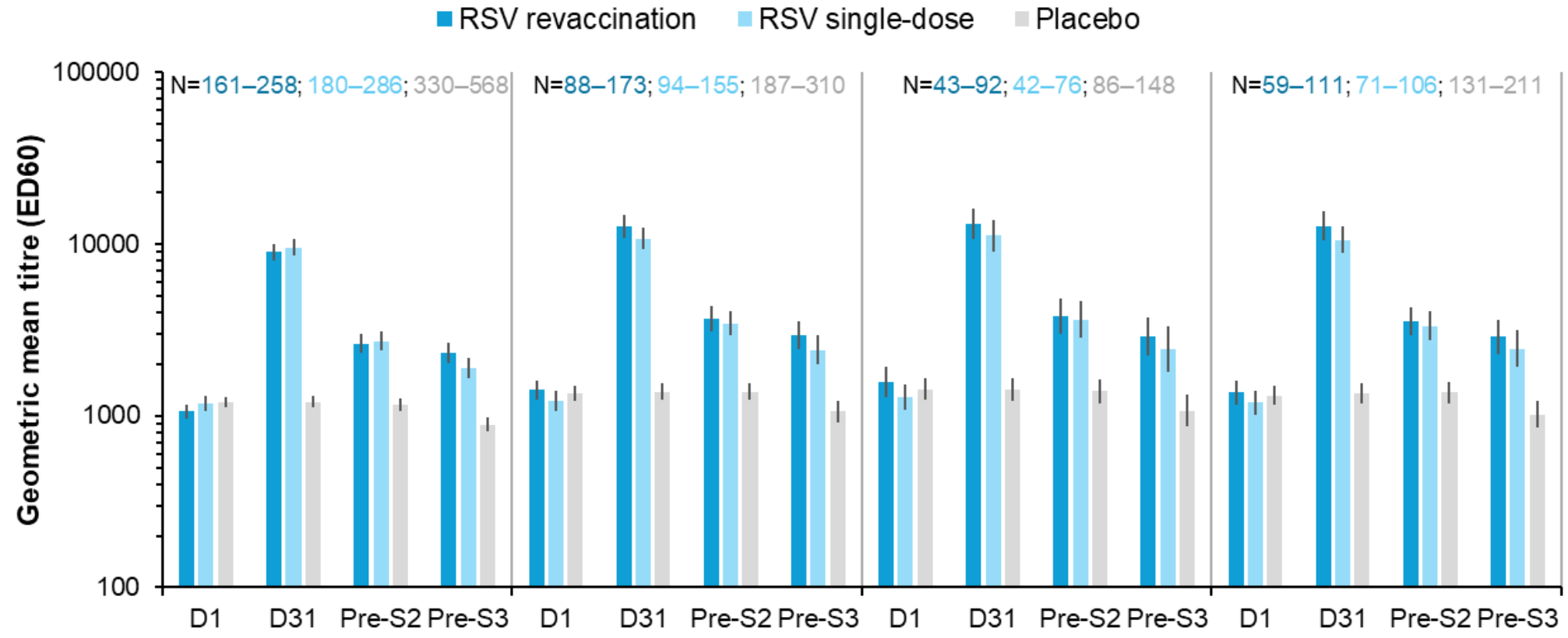

MGI vs D1 (95% CI)		No condition	≥1 condition	≥1 cardiorespiratory condition	≥1 endocrine/metabolic condition
RSV revaccination	D31	8.5 (7.5–9.5)	9.1 (7.7–10.7)	8.4 (6.6–10.7)	9.3 (7.5–11.7)
	Pre-S2	2.6 (2.3–2.9)	2.6 (2.2–3.1)	2.4 (1.9–3.1)	2.6 (2.1–3.3)
	Pre-S3	2.4 (2.1–2.7)	2.4 (2.0–2.8)	2.1 (1.6–2.8)	2.4 (1.9–3.0)
RSV single-dose	D31	8.1 (7.2–9.1)	8.9 (7.7–10.4)	8.8 (7.2–10.7)	9.0 (7.4–11.0)
	Pre-S2	2.3 (2.1–2.6)	2.7 (2.4–3.1)	2.7 (2.2–3.2)	2.7 (2.3–3.2)
	Pre-S3	1.6 (1.5–1.8)	1.9 (1.6–2.1)	1.7 (1.5–2.1)	1.9 (1.6–2.3)
Placebo	D31	1.0 (1.0–1.1)	1.0 (1.0–1.1)	1.0 (1.0–1.1)	1.0 (1.0–1.1)
	Pre-S2	1.0 (0.9–1.0)	1.1 (1.0–1.1)	1.0 (0.9–1.1)	1.1 (1.0–1.2)
	Pre-S3	0.8 (0.7–0.8)	0.8 (0.7–0.9)	0.8 (0.7–0.9)	0.8 (0.7–0.9)

Error bars depict 95% CIs. The lower limits of quantification were 18 ED60 for the RSV-A neutralisation assay and 30 ED60 for the RSV-B neutralisation assay.

RSV, respiratory syncytial virus; RSV revaccination, group of participants who received a first dose of adjuvanted RSV prefusion F protein-based vaccine (adjuvanted RSVPreF3) pre-season 1 and a second dose (revaccination) pre-season 2; RSV single-dose, group of participants who received a single dose of adjuvanted RSVPreF3 pre-season 1 and a placebo dose pre-season 2; placebo, group of participants who received placebo pre-season 1 and pre-season 2; N, number of participants with available results (range across timepoints is given per group and per underlying condition subgroup); ED60, estimated dilution 60; D1, day 1 (pre-dose 1); D31, day 31 (1 month post-dose 1); pre-S2, pre-RSV season 2 (pre-dose 2); pre-S3, pre-RSV season 3; MGI, mean geometric increase (i.e., geometric mean of the within-participant ratios of the post-vaccination over pre-vaccination titres); CI, confidence interval.

Supplementary references

1. Papi A, Ison MG, Langley JM, et al. Respiratory Syncytial Virus Prefusion F Protein Vaccine in Older Adults. N Engl J Med 2023; 388:595-608.
